# Targeting the tripartite axis of immune-metabolic-spatial crosstalk to overcome therapy resistance in breast cancer

**DOI:** 10.3389/fimmu.2026.1748257

**Published:** 2026-03-23

**Authors:** Siyu He, Xuyan Liu, Qingjie Lv

**Affiliations:** 1 Department of Pathology, Shengjing Hospital of China Medical University, Shenyang, Liaoning, China; 2 Key Laboratory of Intelligent and Precision Pathology Diagnosis in Oncology, China Medical University, Shenyang, Liaoning, China; 3 Department of Pathology, Liaoning Health Industry Group Bengang General Hospital, Benxi, Liaoning, China

**Keywords:** breast cancer, drug resistance, immune regulation, metabolic reprogramming, tumor microenvironment

## Abstract

Therapeutic resistance remains the principal cause of mortality in breast cancer. While the tumor microenvironment (TME) is a key contributor, therapies targeting isolated TME components, whether immune, metabolic, or spatial, have largely failed due to compensatory adaptations and ecological resilience. This review synthesizes recent advances to propose a tripartite “Immune-Metabolic-Spatial” axis as the fundamental organizer of a robust resistance niche. We elucidate how immunosuppressive cells, such as TAMs and Tregs, are metabolically sustained by altered nutrient availability like lactate and hypoxia, while spatial constraints, including CAF-deposited ECM and DDR1-mediated collagen alignment, physically impede drug delivery and immune infiltration. Critically, we highlight reciprocal crosstalk where metabolic reprogramming dictates immune cell function, in turn influencing stromal remodeling to create a self-reinforcing resistance loop. Beyond mechanism, we evaluate emerging strategies that concurrently target multiple axes, such as combining immune checkpoint blockade with metabolic inhibitors or stromal disruptors. Finally, we discuss clinical translation through biomarker development and innovative trial designs, framing the tripartite axis as an actionable framework for overcoming therapeutic resistance.

## 1 Introduction

Therapeutic resistance represents the foremost challenge in the clinical management of breast cancer, ultimately driving disease recurrence and mortality (1). While decades of research have yielded targeted therapies and immunotherapies that significantly improve outcomes, their benefits are still transient (2). Tumors invariably develop evasion mechanisms, highlighting a fundamental inadequacy in our current therapeutic paradigms (3). Compounding this challenge, current diagnostic approaches—including imaging and tissue biopsy—often detect disease at advanced stages when resistance mechanisms are already entrenched. Emerging liquid biopsy technologies based on surface-enhanced Raman scattering (SERS) offer the potential for non-invasive early detection, as recently demonstrated using novel porous silicon Bragg reflector substrates that achieved 95% diagnostic accuracy in distinguishing early breast cancer patients from healthy controls (4).

This inadequacy stems from a historical, yet persistent, reductionist approach to the tumor microenvironment (TME). Extensive research has meticulously characterized individual TME components—immunosuppressive cell populations (5), metabolic reprogrammin (6), and desmoplastic spatial architecture (7)—and their respective contributions to therapy failure. However, clinical strategies targeting these axes in isolation, such as immune checkpoint monotherapy or metabolic inhibition, have met with limited success (8, 9). A critical consensus is emerging: the TME operates not as a collection of discrete parts, but as a complex, adaptive ecosystem (10). Its resilience is derived from profound cross-dimensional compensation; targeting one axis merely invites evasion through another, leaving the core resistant niche intact (11). To breach this ecological barrier, a new integrative framework is urgently needed.

**Figure 1:**
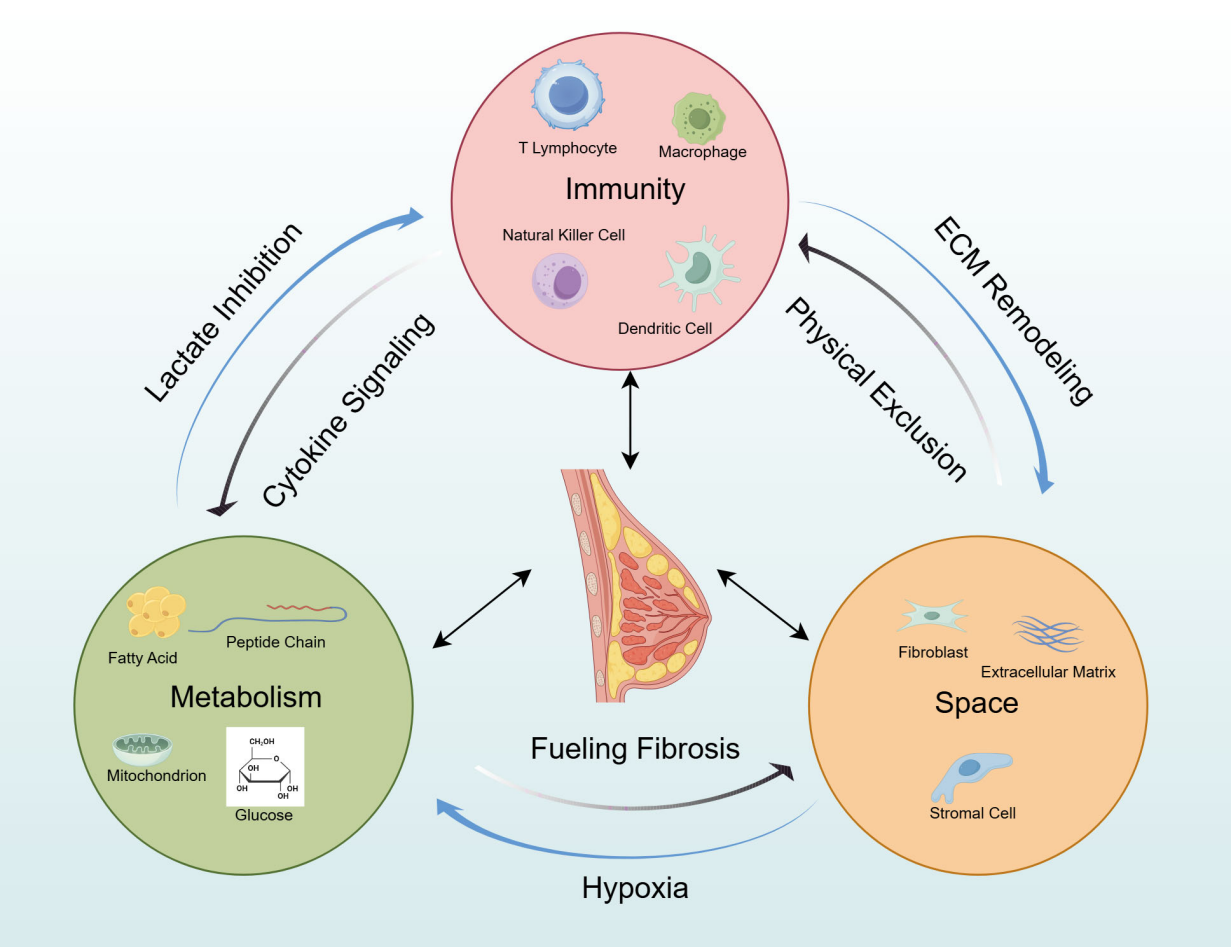
The immune-metabolic-spatial tripartite axis collaboratively fosters a therapy-resistant niche in breast cancer. This schematic illustrates how three interconnected dimensions within the tumor microenvironment collectively drive therapy resistance. Immunosuppression (e.g., Tregs, M2-TAMs), metabolic reprogramming (e.g., lactate, hypoxia), and spatial heterogeneity (e.g., CAFs, collagen alignment) engage in complex bidirectional crosstalk, synergistically establishing a protective niche. This niche not only restricts the infiltration and function of cytotoxic T cells but also compromises the delivery and efficacy of therapeutic agents, ultimately leading to immune evasion and treatment failure.

This review posits that therapeutic resistance in breast cancer is orchestrated by a tripartite “Immune-Metabolic-Spatial” axis, a synergistic network that functions as the fundamental organizer of the resistant niche. We move beyond a siloed discussion to elucidate how these dimensions engage in reciprocal crosstalk: how metabolic dysregulation impairs immune effector function, how spatial constraints dictate metabolic and immune responses, and how immune cells actively remodel the stromal and metabolic landscape. The objective of this review is threefold. First, we decrypt the molecular mechanisms that underpin the dialogue within this tripartite axis. Second, we examine how their integration fosters resistance across breast cancer subtypes. Finally, and most critically, we translate these insights into a discussion of rational combinatorial therapeutic strategies designed to concurrently disrupt multiple axes. By framing the TME as an interconnected whole, we aim to provide an actionable blueprint for overcoming therapeutic resistance and advancing the next generation of immunotherapy-based breast cancer treatments (**Figure 1**). Ultimately, by dissecting this tripartite axis from an immunological perspective, this review seeks to illuminate novel co-targeting strategies that can reinvigorate antitumor immunity and break the vicious cycle of treatment failure in breast cancer.

Despite significant advances in dissecting individual components of the tumor microenvironment, the mechanisms by which immune dysfunction, metabolic reprogramming, and spatial heterogeneity cooperatively drive therapeutic resistance remain poorly defined. This review synthesizes emerging evidence to address four fundamental questions at the intersection of these dimensions:

First, how do immunosuppressive networks, metabolic adaptations, and physical barriers mechanistically intersect to establish a self-reinforcing resistant niche? While each axis has been extensively characterized in isolation, their synergistic crosstalk—and the molecular nodes that integrate signals across dimensions—remains incompletely understood.

Second, which rate-limiting hubs within this tripartite network represent the most clinically actionable targets? Identifying nodes that simultaneously modulate immune, metabolic, and spatial compartments is essential for rational design of combination therapies that preempt compensatory resistance mechanisms.

Third, what is the current clinical evidence for multi-axis targeting strategies, and how can emerging biomarkers guide patient stratification? We critically evaluate ongoing trials and discuss the translational challenges—including trial design, toxicity management, and endpoint selection—that must be addressed to realize the promise of integrated approaches.

Fourth, how does the immune-metabolic-spatial landscape differ across organ-specific metastatic sites, and what therapeutic opportunities arise from these differences? The distinct microenvironments of lung, brain, bone, and liver metastases demand organ-tailored strategies that account for local metabolic constraints and immune privilege.

By systematically addressing these questions, this review aims to transform the tripartite axis concept from a descriptive framework into an actionable roadmap for developing next-generation therapies that durably dismantle the resistant niche in breast cancer.

## 2 Immune microenvironment: from cellular interactions to molecular regulation

The immune microenvironment of breast cancer constitutes a dynamically balanced ecosystem where myeloid and lymphoid cells collaboratively shape an immunosuppressive niche through intricate network interactions (12, 13). Recent single-cell sequencing and spatial transcriptomic studies have uncovered that these immune cells not only exhibit functional heterogeneity but also form multilayered resistance barriers via their spatial distribution patterns and molecular regulatory hubs (14).The myeloid-derived immunosuppressive landscape in breast cancer comprises three major interconnected populations: myeloid-derived suppressor cells (MDSCs), M2-polarized tumor-associated macrophages (TAMs), and dendritic cells (DCs). Rather than functioning in isolation, these populations engage in dynamic crosstalk to establish a self-reinforcing immunosuppressive network. In this section, we first discuss how MDSCs orchestrate immune evasion through metabolic competition and cytokine secretion. We then examine how M2 macrophages sustain this immunosuppressive milieu while also promoting tumor stemness and metastasis. Finally, we highlight the dual role of DCs—whose antigen-presenting function is often compromised in the TME—and emerging strategies to restore their immunostimulatory capacity. Understanding the interplay among these myeloid subsets is essential for designing effective combination immunotherapies.

### 2.1 Myeloid-derived immunosuppressive network

Myeloid-derived immunosuppressive cells play a pivotal role in breast cancer progression and therapy resistance by establishing complex immunosuppressive networks. Emerging evidence highlights their phenotypic plasticity and functional heterogeneity, rendering them dual entities as both tumor promoters and therapeutic targets.

In triple-negative breast cancer (TNBC), the MCT-1 gene promotes macrophage polarization toward the M2 phenotype through the IL-6/sIL-6R signaling axis, significantly enhancing tumor invasiveness and stem-like traits. Notably, targeting this pathway—via IL-6R blockade or MCT-1 silencing—not only reverses M2 polarization but also induces antitumor M1-like macrophages by upregulating miR-34a (15). Post-neoadjuvant chemotherapy, interferon-activated macrophages exert dual effects via a unique IRENA-mediated mechanism. IRENA, a critical regulator, bridges protein kinase R (PKR) and NF-κB signaling to facilitate protumor factor secretion. Clinical data demonstrate that high IRENA expression correlates with poor treatment response. Importantly, macrophage-specific IRENA ablation eliminates its pro-resistance function without impairing antitumor immunity, offering a novel rationale for combination therapies targeting myeloid cells (12). In therapeutic innovation, chimeric antigen receptor macrophage (CAR-M) therapy has shown promise. The first clinical trial of anti-HER2 CAR-M confirmed that engineered macrophages effectively remodel the tumor microenvironment, achieving a 44% disease stabilization rate at eight weeks post-treatment in HER2 3+ patients (16).

Macrophages also foster metastasis by recruiting neutrophils to establish a prometastatic niche. Studies reveal that macrophage-induced physical interactions between neutrophils and tumor cells (PICs) significantly enhance tumor invasion and angiogenesis. Patients with advanced breast cancer exhibiting high PICs signatures have poorer survival, suggesting this interaction network as a potential therapeutic target (17). Conventional dendritic cells (cDCs), particularly the DC1 subset, regulate chemotherapy immunogenicity through the ANXA1/FPR1 axis. ANXA1 released by dying cancer cells binds to FPR1 on cDCs, promoting their migration toward apoptotic tumor cells and facilitating antigen cross-presentation to T cells. Approximately 30% of individuals carry a loss-of-function FPR1 variant (rs867228), which compromises this interaction, reduces anthracycline response, and elevates breast cancer risk—particularly for the luminal B subtype. The TLR3 ligand polyinosinic:polycytidylic acid (pIC) restores chemotherapy sensitivity in FPR1-deficient models by enhancing DC- and T cell-mediated antitumor immunity, improving tumor regression rates (18).

Future studies should focus on deciphering breast cancer subtype-specific myeloid heterogeneity, elucidating the dynamic evolution patterns of myeloid cells induced by therapies, and developing multi-omics-integrated precision targeting strategies. These breakthroughs will be crucial for overcoming myeloid cell-mediated drug resistance barriers. While myeloid cells establish the immunosuppressive foundation of the TME, the adaptive and innate lymphoid populations—particularly T cells and NK cells—serve as the ultimate executors of antitumor immunity. Their dysfunction represents a critical barrier to therapeutic success, as discussed in the following section.

### 2.2 Lymphoid-derived immunosuppressive networks

The lymphoid compartment of the breast cancer immune microenvironment comprises two major interconnected populations: T lymphocytes and natural killer (NK) cells. While T cells orchestrate adaptive immune responses through antigen-specific recognition, NK cells provide critical innate immunosurveillance. Both populations are subject to multifaceted immunosuppressive mechanisms that drive therapy resistance. In this section, we first discuss how CD8^+^ and CD4^+^ T cell functions are regulated by epigenetic, metabolic, and signaling pathways in triple-negative breast cancer (TNBC). We then examine emerging evidence on T cell-based engineered therapies. Finally, we highlight the unique roles of NK cells across breast cancer subtypes and strategies to restore their cytotoxicity. Understanding the interplay between these lymphoid populations is essential for developing effective immunotherapies. In TNBC, T cell function is precisely regulated through multiple mechanisms. At the epigenetic level, ARID1A (AT-rich interaction domain 1A)—a tumor suppressor gene and core component of the SWI/SNF (BAF) chromatin-remodeling complex that regulates DNA packaging and gene expression—significantly suppresses CD8^+^ T cell function via the NPM1-PD-L1 axis (13), with clinical data showing that patients with low ARID1A expression exhibit greater sensitivity to programmed cell death protein 1 (PD-1) inhibitor therapy (13). Concurrently, nuclear AURKA, aurora kinase A, a serine/threonine kinase involved in cell cycle regulation and mitotic spindle assembly, has been found to enhance immune responses by activating CD8^+^ T cell proliferation and activity while upregulating PD-L1 expression through a MYC-independent pathway (19). Recent spatial analyses reveal that CD39^+^ tissue-resident memory CD8^+^ T cells possess trans-site clonal expansion capabilities (20), and PD-1 blockade effectively restores their proliferative potential (20). Tumor cells downregulate immune gene expression via the integrin αvβ6-TGFβ-SOX4 axis, leading to T cell resistance, targeting αvβ6 restores TNBC cell sensitivity to cytotoxic T cells and PD-1 therapy (21).The MTDH-SND1 complex—comprising metadherin (MTDH) and staphylococcal nuclease domain-containing protein 1 (SND1), an oncogenic complex that promotes mRNA degradation—weakens tumor immunogenicity by degrading TAP1/TAP2 mRNA (transporter associated with antigen processing 1 and 2), which are critical for peptide loading onto MHC class I molecules, thereby inhibiting antitumor T cell activity (22). Targeting this complex also significantly enhances PD-1 inhibitor efficacy, providing a promising target for novel combination therapies. These discoveries offer new intervention targets to overcome immune checkpoint resistance. In engineered therapies, T cell receptor-engineered T cell (TCR-T) cells targeting the TNBC-specific antigen ROPN1 —a cancer-testis antigen involved in cytoskeletal dynamics that is selectively expressed in triple-negative breast cancer but absent in normal adult tissues except testis—demonstrate precise cytotoxic potential (23), opening new avenues for personalized immunotherapy. In CD4^+^ T cells, TGF-β signaling promotes resistance by suppressing T helper type 2 cell (TH2) responses; the bispecific decoy molecule 4T-Trap targeting this pathway, when combined with vascular endothelial growth factor (VEGF) inhibitors, enhances antitumor effects (24). Notably, CDK4/6 inhibitors promote CD8^+^ T cell differentiation toward memory phenotypes via the MXD4-MYC pathway (25). Additionally, MHC-II-restricted antigen presentation mediates B cell-CD4^+^ T cell collaboration, generating potent antitumor immunity (26). These studies provide novel insights for developing combination immunotherapies.

Beyond adaptive T cell responses, innate lymphoid cells—particularly natural killer (NK) cells—play unique and complementary roles in breast cancer immunosurveillance. Recent studies have uncovered multifaceted mechanisms regulating NK cell function across different breast cancer subtypes. NK cells play unique roles in breast cancer immunosurveillance through multifaceted mechanisms. Recent studies show that ProkR2-expressing sensory neurons in the sciatic nerve activate NK cell function via the D1-like dopamine receptor-cAMP-PKA-CREB pathway. This neural stimulation also induces PD-L1 expression through interferon-gamma (IFN-γ), synergizing with anti-PD-1 therapy (27). In human epidermal growth factor receptor 2-positive (HER2^+^) breast cancer, human leukocyte antigen-G (HLA-G) drives trastuzumab resistance by engaging the NK cell receptor KIR2DL4—this aberrantly activates a TGF-β/IFN-γ feedback loop that coordinately upregulates PD-L1/PD-1 expression to suppress NK cell function. Blocking HLA-G significantly improves therapeutic sensitivity (28). Clinically, CAR-NK cells (NK-92/5.28.z) demonstrate sustained cytotoxicity in HER2^+^ models (29). Epigenetically, the PRC1 component RNF2 regulates NK cell activation, and its knockout markedly enhances antitumor activity (30). In estrogen receptor-positive (ER^+^) breast cancer, targeting the circTNK2-STAT3-CXCL10 axis during endocrine therapy resistance increases CD56^+^ NK cell infiltration, reversing resistance (31). Dormant ER^+^ breast cancer stem cells evade NK cell killing via BACH1/SOX2-mediated transcriptional programs (BACH1: BTB domain and CNC homolog 1, a transcription factor that regulates oxidative stress responses; SOX2: SRY-box transcription factor 2, a stemness-associated transcription factor), which STING agonists can overcome (32). These findings collectively reveal the diverse regulation of NK cells across breast cancer subtypes.

Lymphocyte interactions within the tumor microenvironment form a complex regulatory network. ALECSAT-generated polyclonal lymphocytes combined with PD-L1 blockade exhibit synergistic therapeutic effects (33), offering a new adoptive cell therapy option. Single-cell sequencing analyses reveal that TNBC’s regulatory T cells, exhausted CD8^+^ T cells, and B cells form a distinct immunosuppressive network (34), providing fresh perspectives on therapy resistance mechanisms. As pivotal immune regulatory hubs (35), tumor-draining lymph nodes exert direct influence on CD8^+^ T cell infiltration and macrophage polarization states through their structural and functional integrity. Cutting-edge research on T/NK cell regulation and lymphocyte crosstalk has uncovered intricate immune mechanisms, offering significant implications for developing novel immunotherapies and combination strategies while charting new research directions.

### 2.3 Interplay between diverse immune cell populations

In breast cancer immunotherapy, dynamic interactions among distinct immune cell types have been established as pivotal determinants of therapeutic response. Research has identified the crosstalk between CXCL13+ T cells and pro-inflammatory macrophages as a predictive biomarker for combination therapy efficacy, with its presence significantly correlating with improved clinical outcomes (36). Paclitaxel monotherapy was found to deplete crucial immune populations, including CXCL13+ T cells and follicular B cells, while the PD-L1 inhibitor combination could counteract this immunosuppressive effect (36). Single-cell analyses revealed that patients with limited baseline immune infiltration, following PD-1 inhibitor plus radiotherapy, exhibited marked immune activation signatures characterized by functional interactions between cytotoxic T cells and specific myeloid subsets (37). Mast cells emerge as key regulators in the TNBC microenvironment, with antigen-presenting mast cells (apMCs) capable of effectively activating T cell responses. Clinical studies demonstrate that sodium cromoglicate (targeting apMCs) combined with PD-1 inhibitors achieved a 50% objective response rate (38). Tumor-associated macrophage (TAM)-derived IL1β upregulates PD-L1 expression via the IL1R2/YY1/c-Fos axis, promoting CD8^+^ T cell exhaustion. IL1R2 blockade synergized with anti-PD-1 therapy to concurrently reduce TAM infiltration, suppress breast tumor-initiating cell (BTIC) self-renewal, and restore T cell function (39). These findings underscore the therapeutic potential of targeting immune cell interactions in combination with PD-1/PD-L1 blockade.

In ER+ breast cancer, invasive ductal carcinoma (IDC) exhibits macrophage-T cell interactions associated with prolonged disease-free survival, whereas invasive lobular carcinoma (ILC) lacks this protective immune crosstalk pattern (40). The immunogenic subtype of HR+/HER2- breast cancer, enriched with immune cell infiltration, may derive benefit from checkpoint inhibition (41), contrasting sharply with RTK-driven subtypes. For HER2+ treatment resistance, the novel CD47/HER2 bispecific antibody IMM2902 demonstrates breakthrough efficacy by stimulating macrophage secretion of CXCL9/10, thereby enhancing T/NK cell recruitment. This agent shows superior antitumor activity across multiple resistant models, including patient-derived xenograft (PDX) compared to conventional therapies (42).In TNBC, NAC1 maintains cancer stemness while modulating myeloid-derived suppressor cell (MDSC) function to establish immunosuppressive niches, with its tumorigenicity markedly enhanced in NK cell-deficient models (43). Tumor cell CD28 directly binds Cd274 mRNA and recruits splicing factor SNRPB2 to stabilize PD-L1 transcripts, resulting in CD8+ T cell dysfunction and reduced cDC1 infiltration (44). Gut microbiome analyses reveal that “therapy-refractory breast cancer” subtypes harbor higher mutational burdens and more complex immune microenvironments, with scoring indices inversely correlating with treatment response and immune cell abundance (45).

Current research elucidates immune cell interactions across breast cancer subtypes including TNBC, ER+, hormone receptor positive(HR+) breast cancer, establishing their associations with treatment response and prognosis. These findings highlight the considerable potential of combining immune checkpoint inhibitors with strategies targeting intercellular crosstalk networks. Building upon these insights into immune cell crosstalk, the molecular signaling pathways that orchestrate these interactions represent the next layer of complexity. The following section examines how these intracellular networks integrate tumor-intrinsic and immune-regulatory mechanisms to shape therapeutic responses.

### 2.4 Molecular signaling pathways regulating immune hubs

The immunomodulatory landscape of breast cancer is governed by intricate molecular signaling networks that integrate tumor-intrinsic mechanisms with immune cell functionality to shape therapeutic responses. Chemotherapy-surviving tumor cells activate multilayered immunoregulatory programs, such as enhancing chromatin accessibility to promote IRF1-dependent interferon response gene expression or upregulating CD80 via p53 signaling, thereby establishing redundant immunosuppressive barriers. Studies demonstrate that even combined targeting of PD-L1 and CD80 fails to completely eradicate these residual lesions with multilayered protective mechanisms, underscoring the need for more precise intervention strategies (46). The subcellular localization of PD-L1 represents a critical determinant of immunotherapy response. In paclitaxel-treated tumors, the ATAD3A-PINK1 axis regulates PD-L1 distribution patterns through mitophagy modulation—membrane-localized PD-L1 facilitates immune evasion while mitochondrial localization promotes its degradation. Preclinical studies confirm that ATAD3A targeting not only reprograms PD-L1 distribution but also significantly remodels the immune microenvironment, offering novel approaches to overcome resistance in chemo-immunotherapy combinations (14).

The interferon signaling pathway serves as a pivotal nexus connecting innate and adaptive immunity. UBA7-mediated ISGylation facilitates STAT1/2 nuclear relocation to activate chemokine networks and enhance cytotoxic T cell infiltration. Notably, age-related interferon signaling attenuation in elderly patients’ tumors can be reversed by STING agonists, providing a rationale for personalized combination therapies (47, 48). Concurrently, the LCOR protein regulates antigen presentation machinery independently of interferon signaling, with its loss driving immune evasion in tumor stem cells. Innovative exosome-delivered LCOR-mRNA has proven effective in restoring tumor sensitivity to PD-L1 blockade (49).

Epithelial mesenchymal transition (EMT) processes are intimately linked with immunosuppressive microenvironment formation. Research reveals that rare mesenchymal-featured tumor cells can establish protective niches through secreted factors (CD73, CSF1), conferring immunotherapy resistance to the entire tumor population. This “cross-protection” effect diminishes significantly upon CD73 blockade, suggesting EMT-related immunomodulators as promising combination targets (50). In genetically high-risk populations, BRCA1 mutation-induced epigenomic dysregulation drives luminal progenitor malignant transformation via EMT while triggering early immunosuppressive signaling, creating a preventive intervention window (51). Cytokine networks exhibit pathway-specific regulatory patterns in breast cancer. The IL6/STAT3 axis activates metastasis programs independently of estrogen receptor signaling in ER+ tumors and remains unresponsive to endocrine therapy. Selective STAT3 inhibition markedly reduces tumor invasiveness, with this ER pathway uncoupling phenomenon informing new targeted strategy design (52). Antibody-drug conjugates (ADCs) demonstrate unique advantages in breast cancer treatment. Phase III trials confirm that the novel ADC ARX788 achieves a median progression-free survival (mPFS) of 11.3 months in HER2+ breast cancer, significantly outperforming conventional regimens (53). With 14 approved and over 100 investigational ADCs, these agents are reshaping treatment paradigms, though predictive biomarkers are urgently needed for patient stratification (54). Recent molecular subtyping of HR+/HER2+ breast cancer identified four subtypes, including an immunomodulatory variant with ADC sensitivity (55), potentially refining beneficiary selection.

These molecular insights not only deepen our understanding of breast cancer immunobiology but also provide multi-layered therapeutic targets to overcome current treatment limitations. From precision control of PD-L1 localization to rejuvenating aged immune systems, from enhanced antigen presentation to reversing EMT-associated immunosuppression, future combination strategies must integrate these pathway interactions to achieve durable therapeutic efficacy.

The immune mechanisms discussed above—TAM polarization, T cell exhaustion, MDSC expansion—are not standalone phenomena. Within the tripartite framework, they function as convergence points where metabolic reprogramming and spatial architecture license and amplify immunosuppression.

Consider three representative examples from this section. The MCT-1/IL-6 axis driving M2 polarization in TNBC (14) is not merely an immune-polarization pathway—it is a direct link between tumor glycolysis (metabolic axis) and macrophage phenotype (immune axis), with its activity spatially restricted to lactate-rich, hypoxic niches. The MTDH-SND1 complex, which degrades TAP1/TAP2 mRNA to impair antigen presentation (21), represents a point where tumor-intrinsic metabolic stress intersects with immune evasion, and its efficacy as a target depends on the spatial distribution of antigen-presenting cells within the TME. Similarly, TAM-derived IL1β promoting CD8^+^ T cell exhaustion (38) operates within a spatially defined niche where physical barriers (dense ECM) and metabolic competition (arginine depletion) converge to create a self-sustaining immunosuppressive loop.

This integrative lens yields three insights unavailable from immune-centric analyses. First, targeting immune checkpoints alone fails because metabolic support (e.g., lactate-fueled Treg stability) and physical barriers (e.g., CAF-deposited collagen excluding T cells) remain intact. Second, the same immune mechanism exerts context-dependent effects—CD8^+^ T cells in perivascular niches behave differently from those trapped in dense stroma. Third, the most clinically actionable targets reside at the intersection of all three axes, such as ENPP1, which simultaneously modulates adenosine metabolism (metabolic), myeloid infiltration (immune), and neutrophil extracellular trap formation (spatial), or DDR1, whose disruption of collagen alignment (spatial) restores T cell access (immune) and normalizes metabolic gradients.

|By framing immune mechanisms as embedded within this tripartite network, our analysis transforms descriptive cataloging into a mechanistic roadmap for multi-axis intervention. The immune populations reviewed here are not isolated drivers of resistance but integral components of a dynamic, cross-dimensional ecosystem—a distinction that fundamentally shapes the combination strategies proposed in subsequent sections.

## 3 Metabolic reprogramming: how metabolites reshape immunity and spatial organization

Metabolic reprogramming has emerged as a hallmark of cancer that not only fuels tumor growth but also actively shapes the immune landscape and spatial architecture of the tumor microenvironment (TME). Breast cancer cells exhibit remarkable metabolic plasticity, adapting their bioenergetic and biosynthetic pathways to sustain proliferation, survive therapeutic stress, and evade immune surveillance. Beyond cell-autonomous effects, metabolites such as lactate, reactive oxygen species (ROS), and amino acid derivatives function as signaling molecules that modulate immune cell function and stromal remodeling. In this section, we examine three interconnected layers of metabolic reprogramming: energy metabolism switching (including oxidative phosphorylation and glycolysis), hypoxia-driven metabolic adaptation, and subtype-specific metabolic vulnerabilities. Understanding how these metabolic nodes intersect with immune and spatial dimensions is essential for developing effective combination therapies. The metabolic adaptability of breast cancer cells is closely associated with therapeutic resistance. In TNBC, TMEM65 acts as a novel oncogene that promotes tumor progression by enhancing mitochondrial oxidative phosphorylation (OXPHOS) and ROS production. MYC and TET3 cooperatively upregulate TMEM65 expression, which then enhances tumor stemness through the HIF1α-SERPINB3 axis, a signaling pathway in which HIF1α (hypoxia-inducible factor 1-alpha), a transcription factor stabilized under hypoxic conditions, upregulates SERPINB3 (serpin family B member 3), a serine protease inhibitor with anti-apoptotic and pro-stemness functions, explaining the molecular mechanism of cisplatin resistance in TNBC (56). Notably, chemotherapy-resistant TNBC cells exhibit a clear metabolic preference shift toward OXPHOS dependence, making them sensitive to specific OXPHOS inhibitors and providing new strategies to overcome traditional chemotherapy resistance (57). Beyond OXPHOS regulation, metabolic reprogramming can also be driven by degradation of TCA cycle enzymes. The cancer-testis gene MAEL promotes aerobic glycolysis and breast cancer progression by facilitating chaperone-mediated autophagy (CMA)-dependent degradation of citrate synthase (CS) and fumarate hydratase (FH), two key enzymes of the TCA cycle (58). Mechanistically, MAEL interacts with CS/FH via its MAEL domain and with HSPA8 via its HMG domain, enhancing the binding affinity of CS/FH to HSPA8 and their transport to lysosomes for degradation. This leads to a metabolic shift from oxidative phosphorylation to glycolysis, upregulation of HIF-1α and its target genes (VEGF, Cyclin D1, Snail), and downregulation of E-cadherin—effects reversible by CS/FH overexpression. This mechanism represents a direct link between protein degradation pathways (autophagy) and metabolic reprogramming, illustrating how post-translational regulation of metabolic enzymes contributes to the Warburg effect in breast cancer.Reprogramming of glucose metabolism pathways shows significant subtype specificity in breast cancer. CDK5 activates G6PD through Thr-91 phosphorylation, redirecting glucose metabolism from glycolysis to the pentose phosphate pathway (PPP) to help tumor cells maintain redox balance. Preclinical studies demonstrate that targeting the CDK5-G6PD axis not only inhibits tumor growth but also enhances the efficacy of PARP inhibitors (59). In TNBC, an iridium (III) complex probe targeting the mitochondrial HK2-VDAC1 interaction works through a dual mechanism: inhibiting glycolysis while promoting Bax-VDAC1 binding and ROS production, significantly improving anticancer effects (60).

Hypoxia, a hallmark feature of solid tumors, influences tumor progression through multi-layered metabolic reprogramming. Studies reveal that under hypoxic conditions, circWSB1 transcriptionally activated by HIF1α competitively binds to the deubiquitinase ubiquitin specific peptidase 10 (USP10), blocking its protective effect on p53 and leading to p53 degradation that drives tumor development. This discovery reveals a new mechanism by which hypoxia regulates tumor suppressor pathways through non-coding RNAs (61). Regarding radiotherapy resistance, the sialyltransferase ST3GAL4 promotes endoplasmic reticulum localization of HSP90B1 through catalyzing its sialylation, thereby activating the PERK-EIF2α-ATF4 pathway and upregulating antioxidant factors, ultimately leading to radiotherapy resistance in TNBC. Sialic acid analogs targeting this process can effectively overcome radiotherapy resistance (62).

Different breast cancer subtypes exhibit unique metabolic vulnerabilities. In HER2-positive breast cancer, PPARG activates the PI3K/Akt/mTOR pathway by promoting fatty acid metabolism, leading to anti-HER2 drug resistance. Inhibiting fatty acid synthesis or directly targeting PPARG can restore drug sensitivity, supporting combination strategies of metabolic intervention with anti-HER2 therapy (63). For hormone receptor-positive breast cancer, fasting mimicking diet (FMD) enhances treatment efficacy through multiple mechanisms: reducing IGF1, insulin, and leptin levels, upregulating EGR1 and PTEN expression, and inhibiting the AKT-mTOR pathway, thereby improving endocrine therapy sensitivity and reversing acquired resistance (64). The synergistic induction of ferroptosis and disulfideptosis represents a new direction in metabolic targeting therapy. Research shows that under glucose deprivation conditions, breast cancer cells with high SLC7A11 expression become resistant to ferroptosis but susceptible to disulfideptosis. Based on this, the developed Fe-Cu-SS metal-organic framework can simultaneously induce both death modes: promoting ferroptosis while limiting glucose uptake via BAY876 to cause NADPH depletion-induced disulfideptosis. This dual mechanism demonstrates significantly enhanced therapeutic effects in both *in vitro* and *in vivo* experiments (65).

Current research has revealed the hierarchical network of metabolic reprogramming in breast cancer: from energy metabolism switching (OXPHOS/glycolysis balance) to hypoxia response (HIF1α-circWSB1-USP10-p53 axis), from glucose metabolism redirection (CDK5-G6PD-HK2 regulation) to redox regulation (ROS-antioxidant systems). These findings not only clarify the metabolic basis of treatment resistance but also provide multiple actionable targets for intervention. The metabolic mechanisms discussed above operate across a spectrum of causal certainty. Throughout this section, we have highlighted studies that establish causality through genetic or pharmacological intervention—such as TMEM65-driven OXPHOS dependence validated by knockout models (55), CDK5-G6PD axis confirmed by kinase inhibition (57), and ST3GAL4-mediated radioresistance demonstrated by sialic acid analog rescue experiments (60). These represent prioritized therapeutic targets with direct mechanistic validation. In contrast, findings supported primarily by correlative evidence—including metabolic enzyme expression patterns (61) or hypoxia-induced non-coding RNA associations (59)—are presented as hypothesis-generating observations requiring functional validation. This distinction is critical for translating metabolic insights into actionable combination strategies, as causal drivers are more likely to yield therapeutic efficacy when co-targeted with immune or spatial interventions.These metabolic adaptations not only confer therapeutic resistance but also profoundly influence the physical architecture of the TME. As discussed in the following section, the resulting spatial heterogeneity—including extracellular matrix remodeling and physical barriers—further reinforces the resistant niche, creating a self-perpetuating cycle of treatment failure.

## 4 Spatial heterogeneity: physical barriers and cellular localization in drug resistance mechanisms

Physical barriers within the breast cancer microenvironment represent a critical factor contributing to therapeutic resistance. Research has identified a specialized subset of CD16+ fibroblasts in HER2-positive breast cancer that promote drug resistance through a unique mechanotransduction pathway. When trastuzumab binds to CD16, it triggers the SYK-VAV2-RhoA-ROCK-MLC2-MRTF-A signaling cascade, leading to enhanced cellular contractility and extracellular matrix stiffening. This matrix remodeling not only reduces chemotherapeutic drug delivery efficiency but also physically excludes cytotoxic CD8^+^ T cells from contacting tumor cells, creating a dual therapeutic and immune-privileged barrier. Notably, targeting VAV2—a fibroblast-specific effector molecule in this pathway—effectively reverses the profibrotic response, offering a novel therapeutic target to improve antibody drug efficacy (66). Similarly, three-dimensional bioprinted constrained culture models demonstrate that spatial confinement pressure induces the emergence of CD44+-CD133+ stem-like cell populations at the periphery of mammary tumor spheroids, rendering these cells twice as resistant to doxorubicin/tamoxifen. This “interfacial stress-stemness-resistance” relationship can be reversed by modulating mechanotransduction proteins such as YAP and myosin (67).

The spatial heterogeneity of breast cancer influences treatment response across multiple scales. Large-scale imaging mass cytometry (IMC) studies with 352 patient samples, have revealed the complex diversity of cellular composition and tissue architecture in the tumor microenvironment. By simultaneously analyzing the spatial distribution of 35 biomarkers, researchers identified significant correlations between distinct spatial organization patterns and clinical outcomes, providing a new foundation for precision diagnostics beyond traditional histopathological classification (68). Further technological breakthroughs have enabled three-dimensional IMC analysis at single-cell resolution, successfully identifying microenvironmental features undetectable by two-dimensional imaging in breast cancer samples. This approach provides an essential tool for investigating three-dimensional spatial phenomena such as tumor invasion (69). These high-dimensional spatial analysis technologies confirm that the spatial arrangement patterns of tumor cells and stromal cells create unique “niches” that directly affect drug accessibility and efficacy. The spatial barriers discussed above—ECM remodeling and CAF-mediated contractility—do not operate in isolation but intersect with vascular and perfusion barriers to collectively determine therapeutic resistance. Their relative contributions vary by tumor context. In desmoplastic tumors such as HER2+ breast cancer, CD16+ fibroblast-driven ECM stiffening creates a dominant physical barrier that excludes both drugs and immune cells, as evidenced by trastuzumab resistance mediated primarily by matrix remodeling even when vascular perfusion remains intact (64). Conversely, three-dimensional bioprinted models demonstrate that spatial confinement pressure alone—independent of vascular factors—can induce stem-like phenotypes that confer drug resistance (65), suggesting that ECM barriers and perfusion barriers may operate through distinct mechanisms. Importantly, these barriers are often interdependent: CAF-deposited collagen compresses blood vessels to exacerbate hypoxia and reduce perfusion (64), while matrix stiffness itself can impair vascular function and drug penetration (65). Rather than attempting to quantify a universal hierarchy, we emphasize that the dominant spatial barrier is context-dependent—shaped by tumor subtype, stage, and treatment history—and that effective therapeutic strategies must account for this complexity.

In summary, spatial heterogeneity in breast cancer contributes to therapeutic resistance through: (1) physical barriers that impede drug delivery, and (2) protective cellular/matrix spatial organization patterns that create sanctuary niches for tumor cells.

## 5 Three-dimensional combination therapy: the ultimate strategy to break the drug resistance loop

The development of therapeutic resistance in breast cancer involves intricate interactions among immune, metabolic, and spatial three-dimensional networks. Single-dimensional targeted therapies often yield limited efficacy due to compensatory activation mechanisms. In recent years, combination strategies targeting multi-dimensional synergistic effects have demonstrated breakthrough potential, offering novel approaches to overcome drug resistance.

### 5.1 Immune-metabolism interactions

The response to immunotherapy in breast cancer is closely associated with tumor metabolic reprogramming, and this interaction forms a complex regulatory network. Recent studies have revealed that tumor cells reshape the immune microenvironment through various metabolic pathways, providing important targets for developing novel combination therapeutic strategies. This section will systematically elaborate on these immune-metabolic interaction mechanisms and their clinical translational potential.

The metabolic characteristics of the tumor microenvironment profoundly influence the efficacy of immune checkpoint inhibitors. Clinical trials have confirmed that FMD can safely reduce peripheral immunosuppressive cells while enhancing Th1 responses within tumors (70). In HER2-positive breast cancer, PDPN+ cancer-associated fibroblasts suppress NK cell antibody dependent cell mediated cytotoxicity (ADCC) through IDO1/TDO2-mediated tryptophan metabolic disruption, and dual-target inhibitors can reverse this resistance mechanism (71). Notably, glycosylation modification of B7-H4 stabilizes its protein expression to inhibit immunogenic cell death. Targeting glycosylation with NGI-1 enhances dendritic cell phagocytosis and the ability to activate CD8^+^ IFNγ^+^ T cell responses, thereby significantly improving the efficacy of PD-L1 blockade (72). Lactate metabolism represents another key regulatory node. Metal-phenolic coordinated nanocomposites reduce lactate production by inhibiting lactate dehydrogenase (LDH), and when combined with photodynamic therapy, they effectively activate anti-tumor immunity (73). Similarly, ENPP1-mediated cGAMP hydrolysis suppresses the STING pathway, and its inhibitor synergizes with ATM inhibitors to activate innate immune responses (74). In preclinical models, CTLA-4 blockade enhances the metabolic adaptability of intratumoral T cells, with this immune cell-promoting infiltration effect being more pronounced in tumors with low glycolysis (75). These findings reveal a multi-layered interaction network between metabolic enzymes and immune checkpoints.

Gut and intratumoral microbiota regulate immune responses through metabolites. Flaxseed lignans (FL) are metabolized by gut microbiota into enterolactone (ENL), which enhances T cell function by downregulating CD38 while enriching Akkermansia to improve PD-1 inhibitor response (76). However, intratumorally colonized Sphingomonas promotes CCL20 secretion by consuming propionylcarnitine, recruiting Treg cells, and suppressing CD8+ T cell function (77). CCL20 can also activate granulocyte-monocyte progenitor differentiation via CCR6, inducing PMN-MDSC expansion. These MDSCs further increase ALDH+ tumor stem cells through the CXCL2/NOTCH1/HEY1 pathway (78). The CXCR2 antagonist SB225002 can block this pathway to enhance chemosensitivity (78). In terms of intervention strategies, ACSS2 inhibition converts tumor cells into acetate producers,” providing energy substrates for T cells (79), while acetate supplementation significantly enhances T cell effector functions.

Neurotransmitter-like regulatory pathways exist in the tumor microenvironment. N-acetylaspartate (NAA) disrupts immune synapses through PCAF-mediated laminin acetylation, inhibiting NK and CD8+ T cell cytotoxicity (80). Its derivative NAAG promotes PMN-MDSC differentiation via the RIMKLB-NMDAR-p38 axis, and NMDAR antagonists can enhance PD-1 efficacy (81). Notably, IL-15Rα+ TAMs downregulate CX3CL1 by releasing IL-15/IL-15Rα complexes, inhibiting CD8+ T cell infiltration—a process regulated by the non-transcriptional function of HIF-1α (82). IL-15Rα blocking peptides can restore T cell infiltration and also enhance PD-1 efficacy (82). In amino acid metabolism, DHDH-mediated D-xylose depletion suppresses immune response induction, and xylose supplementation significantly improves CD8+ T cell function (83). These findings expand the regulatory dimensions of “immunometabolism.” Beyond amino acid metabolism, epigenetic regulation of drug resistance genes also intersects with metabolic control. In HER2-positive breast cancer, DNA methylation-mediated upregulation of HOXC8 drives Herceptin resistance by enhancing tumor cell proliferation, clonogenicity, and metastasis while suppressing apoptosis, and interference with HOXC8 expression restores drug sensitivity both *in vitro* and *in vivo* (84). This example illustrates how epigenetic-metabolic crosstalk—here, methylation status dictating expression of a resistance-driving transcription factor—contributes to the immune-metabolic axis of therapeutic failure.

Metabolic reprogramming can directly affect genomic stability. PARP inhibitors drive glucose/lipid metabolic reprogramming in macrophages through SREBP-1, and combining them with CSF-1R blockade can activate CD8+ T cell immunity (85). Under glucose deprivation, c-MYC-induced ASS1 promotes purine synthesis by NO-dependent activation of gluconeogenic enzymes. Tumors with high ASS1 expression are resistant to immune checkpoint inhibitors, but purine synthesis inhibitors can restore sensitivity (86). GDP-M inhibits homologous recombination repair by interfering with BRCA2-USP21 interactions, and its combination with PARPi and PD-1 inhibitors activates the STING pathway (87). Epigenetic regulation plays a key role in the metabolic-immune network. FOXM1 phase-separated condensates maintain oncogenic transcription, while AMPK phosphorylation can disrupt their structure and activate innate immunity (88). The NCOR2-HDAC3 complex suppresses IRF1-dependent immune responses (89), and the ZMYND8-cPLA2α-IL27 axis drives HER2-targeted resistance through abnormal lipid metabolism (90). These mechanisms provide new insights for combination targeting.

In summary, immune-metabolic interactions in breast cancer exhibit the following key characteristics: First, tumor cells construct an immune-evasion-friendly microenvironment by reprogramming glycolysis, amino acid metabolism, and lipid metabolism pathways. Second, gut and intratumoral microbiota bidirectionally regulate host immune responses through metabolites. Third, neurotransmitter-like metabolites and epigenetic regulation further increase the complexity of this network. Notably, these discoveries have already led to various innovative treatment strategies, including combination therapies targeting metabolic enzymes and microbiota interventions.

### 5.2 Immune-spatial interactions

The core of treatment resistance in breast cancer lies in the spatial heterogeneity of the tumor microenvironment, which limits therapeutic response through dual mechanisms of physical barriers and immunosuppressive niches. Recent studies reveal that fibroblast-formed structural barriers in HER2-positive breast cancer are key factors in anti-HER2 therapy resistance. These barriers can be effectively disrupted by the pyrotinib-containing NeoPICD regimen, with the mechanism involving restoration of CD8^+^ T cell and M1 macrophage infiltration into the tumor core (91). The development of spatial analysis technologies has provided essential tools for this research. Imaging mass cytometry (IMC) shows that DDR1-mediated parallel collagen fiber alignment forms physical immune exclusion barriers, with clinical samples demonstrating a significant negative correlation between DDR1 expression and T cell infiltration. Neutralizing antibodies targeting the DDR1 extracellular domain can disrupt this organized structure and significantly enhance immunotherapy efficacy (92). Notably, this spatial regulation exhibits distinct subtype specificity: in ER^+^ breast cancer, proximal enrichment of quiescent stromal cells indicates favorable prognosis, while tumor neighborhoods containing mixed fibroblast phenotypes correlate with poor outcomes (93). Concurrently, specific spatial patterns - including perivascular FOLR2^+^ macrophage-mediated CD8^+^ T cell positioning via the CXCL9-CXCR3 axis and the characteristic distribution of LGALS2^+^ dendritic cells in tertiary lymphoid structures - have been confirmed as key spatial biomarkers for predicting immunotherapy response (94–96).

The spatiotemporal dynamic evolution of CAFs forms an important basis for immune escape. Single-cell studies reveal that CAF-S1 contains eight functional subsets, among which ECM-myCAF and TGFβ-myCAF drive immunotherapy resistance by forming CAF-Treg positive feedback loops (97). More challenging is that senescent CAFs restrict NK cell cytotoxicity through secretion of specialized ECM components, with this mechanism showing significant correlation with recurrence risk across multiple breast cancer subtypes (98). Mechanistic studies demonstrate that CAFs induce downregulation of NK cell activation receptors and functional suppression through specific membrane ligand-receptor binding, with clinicopathological analysis confirming that expanded CAF/NK cell contact areas directly correlate with poor patient prognosis (99). To address these challenges, researchers have developed various innovative strategies, the new drug made of CS-GFLG-DAS selectively induces CAF quiescence through cathepsin B-responsive drug release while maintaining stromal integrity, significantly enhancing PD-1 antibody efficacy (100), ultrasound sonoporation combined with stroma normalization therapy precisely modulates tumor mechanical properties, restoring tissue stiffness to physiological levels while improving perfusion efficiency and CD8^+^ T cell infiltration, achieving 100% survival rate and inducing durable immune memory in animal models (101).

Spatial omics-based precision treatment strategies are transforming clinical paradigms. For TNBC, patients with baseline tertiary lymphoid structure (TLS) and MHC-high features respond well to immunotherapy alone, while those lacking these characteristics require combined radiotherapy to induce spatial synergy between cytotoxic T cells and antigen-presenting myeloid cells (37). Imaging mass cytometry further clarifies that the spatial ratio of proliferating CD8^+^TCF1^+^T cells to MHCII^+^ tumor cells is a key predictor of immune checkpoint inhibitor efficacy, whereas focal enrichment of CD15^+^ tumor cells indicates resistance risk (102). At the molecular level, ZNF689 deficiency increases intratumoral spatial heterogeneity and antigen presentation defects by reactivating LINE-1 retrotransposition, with this unique state being sensitized to immunotherapy through combined LINE-1 inhibition (103). Innovations in delivery systems physically overcome spatial limitations, neutrophil drug carriers (NE-GI-LNPs) achieve deep tumor penetration through damage-driven mechanisms, with near-infrared-triggered GSDME-mediated pyroptosis significantly enhancing antitumor immunity (104).

Current clinical translation faces three core challenges: first, unpredictable treatment responses due to varying levels of intratumoral heterogeneity (ITH) necessitate development of multi-region synchronous monitoring technologies (103); second, dynamic conversion of CAF subsets from immunoregulatory to wound-healing phenotypes during treatment requires temporally precise intervention strategies (105), finally, standardized application of cross-platform spatial analysis technologies (such as CycIF/IMC/MIBI) still requires establishment of unified biomarker validation systems (93). These innovations are driving a paradigm shift in breast cancer treatment from “histology-guided” to “spatial ecology-informed” approaches, ultimately improving clinical outcomes. While immune-spatial interactions have received considerable attention, the metabolic dimension of spatial organization is equally critical. The following section examines how metabolic reprogramming and spatial architecture directly intersect to drive therapeutic resistance.

### 5.3 Metabolic-spatial interactions

Beyond the well-characterized immune-metabolic and immune-spatial axes, the direct crosstalk between metabolic reprogramming and spatial architecture represents an emerging dimension of therapeutic resistance. Metabolic enzymes and metabolites not only fuel tumor growth but also actively remodel the extracellular matrix, influence stromal cell function, and create metabolic niches that protect resistant clones. Conversely, spatial constraints—such as hypoxia and nutrient gradients—impose metabolic adaptations on cancer and stromal cells. In this section, we examine the bidirectional relationship between metabolism and spatial organization, highlighting how this axis contributes to metastasis and therapy resistance. Research has revealed that lymphatic endothelial cells spatially regulate the metastatic microenvironment through an RGS5-mediated oxidative stress sensing system. RGS5(+) endothelial cells exhibit distinct metabolic characteristics, attenuated glycolysis and oxidative phosphorylation, and drive lymph node metastasis by promoting cancer cell-endothelial cell adhesion, a process reversible by antioxidants (106). In therapeutic resistance, CAFs establish spatial barriers through serine metabolism, with the MAPK pathway GTPase cascade, particularly MAP2K7, serving as the key mediator. The A-aLDL nanoparticle system delivering artesunate can disrupt serine metabolic homeostasis in CAFs, effectively dismantling these protective barriers to overcome photothermal therapy resistance in TNBC (107).

However, significant gaps remain in our understanding of metabolic-spatial interaction networks. Future research should focus on developing spatially resolved metabolomics technologies, integrating artificial intelligence modeling to systematically map metabolic-spatial interactions, and exploring novel combination strategies targeting the metabolic-spatial axis. These breakthroughs will advance precision oncology into a new era of spatiotemporal metabolic regulation for overcoming breast cancer treatment resistance. Importantly, these metabolic-spatial interactions do not occur in isolation but are intimately linked with immune regulation. The final section of this chapter integrates all three dimensions, examining how immune, metabolic, and spatial networks converge to create self-sustaining resistant niches.

### 5.4 Immune-metabolic-spatial tripartite interactions

The development of therapeutic resistance in breast cancer results not from isolated factors, but rather from the synergistic interplay of immune suppression, metabolic reprogramming, and spatial heterogeneity. Recent studies demonstrate that these three dimensions collectively construct self-sustaining resistant niches through complex cross-talk.

Research reveals that quiescent cancer cells (QCCs) establish unique resistant niches by forming specialized spatial aggregates. These cellular clusters exhibit not only reduced immune infiltration but also enrichment of dysfunctional dendritic cells and exhausted T cells. Notably, this spatial organization closely associates with hypoxia-induced metabolic reprogramming, jointly forming an immunotherapy “reservoir” (108). In metastatic breast cancer, fibrotic microenvironments (FLN) rich in type I collagen provide protective niches for dormant tumor cells. Pro-resolving macrophage-derived mediators effectively inhibit FLN formation while inducing myofibroblast apoptosis (109). TAMs respond to the stiffening fibrotic TME by activating TGF-β-regulated collagen biosynthesis programs, concurrently depleting arginine and secreting ornithine to impair CD8+ T cell function (110). The ENPP1-mediated adenosine metabolic network represents another critical tripartite node. ENPP1-high circulating tumor cells establish recurrence-favorable microenvironments by promoting PMN-MDSC infiltration and neutrophil extracellular trap (NET) formation, with clinical samples confirming elevated ENPP1 and NETs in recurrent tumors (111). Extracellular vesicles (EVs) play pivotal roles in tri-dimensional crosstalk. LMTK3 modulates EV proteome composition, particularly enhancing PSAT1 packaging to influence monocyte differentiation into M2-like macrophages. This long-distance regulation expands the spatial scope of metabolic influence, reshaping the tumor immune microenvironment (112). Beyond tumor-intrinsic EV signaling, cancer-derived small extracellular vesicles (sEVs) can also mediate inter-organ crosstalk that shapes therapy resistance and off-target toxicity. In the context of doxorubicin-induced cardiotoxicity (DOXIC), breast cancer cells secrete sEVs enriched with miR-338-3p that are taken up by cardiomyocytes, exacerbating DOX-induced ferroptosis through targeted degradation of anti-ferroptotic genes CP, SLC7A11, and GPX4 (113). Mechanistically, DOX upregulates METTL3-mediated m^6^A methylation of pri-miR-338 in breast cancer cells, enhancing its maturation, while RBMX selectively packages miR-338-3p into sEVs for intercellular transfer. This example illustrates how epigenetic regulation (metabolic/regulatory axis), sEV-mediated inter-organ communication (spatial axis), and ferroptosis susceptibility (cell death/immune axis) converge to create a multi-dimensional pathogenic network. Notably, dual-functional decoy sEVs engineered to both neutralize DOX and deliver miR-338-3p inhibitor effectively mitigate cardiotoxicity in tumor-bearing mice, demonstrating the therapeutic potential of targeting such tripartite crosstalk. Three-dimensional organoid models provide essential tools for studying this complex network. By simulating tumor-stroma interactions, these models recapitulate key malignant features and resistance mechanisms, offering ideal platforms for drug screening and combination optimization (114).

Understanding this tripartite network informs novel therapeutic strategies. Current evidence suggests multidimensional interventions may more effectively overcome resistance. For instance, drugs targeting QCC cluster structures combined with immunotherapy could improve T cell infiltration (108). Similarly, ENPP1 inhibitors with radiotherapy may prevent local recurrence (111). Future priorities include developing network-targeting combination strategies, establishing precise 3D evaluation systems, optimizing organoid models, and conducting mechanism-informed clinical trials. This tri-dimensional perspective not only deepens understanding of breast cancer resistance but also provides clinical innovation pathways. Systematic dissection of immune-metabolic-spatial networks and corresponding multidimensional interventions may effectively dismantle resistant niches, ultimately improving patient outcomes.

The concept of a self-reinforcing resistance loop, while compelling, warrants methodological clarification. Strictly defined, bidirectional causality across all three axes—where immune dysfunction directly drives metabolic reprogramming, which in turn alters spatial architecture, which then feeds back to amplify immune suppression within a single experimentally validated model—remains rare in the current literature. The examples discussed above illustrate this challenge. Quiescent cancer cells (QCCs) form immunosuppressive niches characterized by reduced immune infiltration, hypoxia, and spatial aggregation (105); however, whether hypoxia causally drives QCC formation, or QCCs actively remodel their metabolic and spatial environment, involves bidirectional relationships not yet fully disentangled. Similarly, ENPP1 promotes PMN-MDSC infiltration and NET formation (108), establishing clear immune-spatial and immune-metabolic links, but direct evidence that NETs reciprocally enhance ENPP1 expression in tumor cells is lacking.

Rather than requiring complete bidirectional validation for every node, we propose that the tripartite framework be understood as a network synergy model, in which individual mechanisms validated for two-axis interactions collectively create a resilient system. For instance, TAMs respond to stiffened ECM (spatial→immune) while depleting arginine to impair T cells (immune→metabolic) (107); CAFs establish serine-mediated spatial barriers while modulating immune exclusion (spatial↔immune) (104); and lactate-enriched niches sustain Tregs while promoting ECM remodeling (metabolic→immune→spatial) (71, 74). Although no single study currently demonstrates complete three-axis bidirectional causality, the convergence of multiple two-axis loops within the same tumor ecosystem produces an emergent property—the self-reinforcing resistant niche—that is greater than the sum of its parts. This network perspective shifts the therapeutic imperative from disrupting a single causal loop to simultaneously targeting multiple interconnected nodes, a strategy increasingly supported by preclinical combination studies (37, 108).

## 6 Metastasis-specific niches: the distal battlefield of three-dimensional regulation

Breast cancer metastasis is not a simple process of cancer cell dissemination, but rather a systemic pathological process shaped by immune-metabolic-spatial three-dimensional regulation. Current research has revealed several key characteristics of this process. First, metastasis exhibits remarkable selectivity and organ specificity. Studies demonstrate that only a very small proportion of tumor cells, <3%, can successfully metastasize, and these cells display distinct molecular characteristics during lymphatic spread (115). This selectivity depends not only on the intrinsic properties of tumor cells but also critically on the “soil” characteristics of the target organ microenvironment. For example, endothelial cells actively guide tumor cell vascular invasion through the SLIT2-ROBO1 axis, while tumor cell-derived double-stranded RNA can activate this endothelial guidance function via TLR3 (116), forming a bidirectionally regulated metastatic niche.

Second, metastatic microenvironments display unique immunometabolic features. In ER+ breast cancer, PDGF-C signaling activates dormant disseminated tumor cells (DTCs) in aged or fibrotic lung tissues, and this activation can be blocked by inhibiting PDGFRα or using PDGF-C blocking antibodies (117). P38MAPKα inhibition (p38i) limits tumor growth by remodeling the immune microenvironment of metastatic lesions, a process dependent on the coordinated action of CD4+ T cells, IFNγ, and macrophages (118). When combined with OX40 agonists, this approach significantly enhances anti-metastatic effects, particularly in patients with high mutational burden. The success of this combination strategy highlights the importance of immune microenvironment regulation in metastatic sites. Notably, the metastatic mechanisms of TNBC are particularly complex. Research shows that NAC1 promotes metastasis by maintaining cancer stem cell properties and regulating MDSC function, while simultaneously participating in the regulation of oncogenic signaling pathways such as CD44-JAK1-STAT3 and immunosuppressive signals including TGFβ and IL-6 (43). ICAM-1 specifically drives epithelial-mesenchymal transition (EMT) and metastasis through activation of the EGFR-JAK1/STAT3 pathway (119). These findings suggest that different breast cancer subtypes may possess distinct metastatic regulatory networks.

In terms of adaptive immunity, the B cell and T cell receptor repertoires in metastatic lesions exhibit co-evolutionary features with the tumor genome, with metastasis-surveilling B cell clones showing specific expansion patterns (120). This discovery provides new insights for developing metastasis intervention strategies based on immune receptor engineering.

### 6.1 Lung metastasis

The formation of breast cancer lung metastasis involves synergistic interactions among immune microenvironment remodeling, metabolic adaptation, and spatial reorganization. Regarding immune regulation, the pre-metastatic lung microenvironment already exhibits characteristic alterations: early infiltration of inflammatory neutrophils and monocytes (121), followed by TREM2+ regulatory macrophages accumulating at metastatic margins to form immune barriers (121). The lung metastasis process is regulated by multiple signaling pathways, including tumor-derived GM-CSF, which promotes Treg differentiation through the STAT5-AHR-PD-L1 axis (122), and the CTSC-PR3-IL-1β pathway-induced NET formation (123). Notably, the obese microenvironment enhances NET production via ROS (124), while chemotherapy-induced NETs activate TGF-β through integrin-αvβ1/MMP9 to promote EMT (125). Lung-resident stromal cells confer tissue-specific suppressive functions to neutrophils through the PTGS2-PGE2 pathway (126).

Metabolic reprogramming serves as a crucial adaptive strategy for lung metastasis. High interstitial aspartate concentrations drive collagen synthesis via the NMDAR-CREB-DOHH axis (127). TDO2+ fibroblasts secrete kynurenine to help tumor cells resist ferroptosis while inducing T cell dysfunction through the CCL8/11-KYN axis (128). Under chemotherapy pressure, circulating tumor cell (CTC) clusters acquire drug resistance through ST6GAL1-mediated desialylation (129), revealing the role of metabolic plasticity in treatment resistance. In terms of spatial organization, intratumoral bacteria enhance CTC mechanical stress resistance (130), and polyclonal CTC clusters achieve immune escape by downregulating NK cell ligands (131).

These findings provide multi-target intervention strategies for lung metastasis treatment: combining approaches targeting SF-Ron to enhance T cell responses (132), inhibiting CTSC to block NET formation (123), or intervening in the aspartate-NMDAR axis (127) and TDO2-KYN pathway (128) may effectively disrupt metastatic niches. Future research should focus on elucidating subtype-specific mechanisms of breast cancer lung metastasis to advance precision therapy. While the lung represents a common metastatic site for breast cancer, the brain poses unique challenges due to its distinct metabolic environment and immune privilege. The following section examines how breast cancer cells adapt to the brain microenvironment to establish metastatic lesions.

### 6.2 Brain metastasis

The formation of breast cancer brain metastasis involves unique metabolic adaptations and microenvironmental interactions. Research reveals that HER2+ breast cancer brain metastatic cells exhibit remarkable metabolic plasticity (133), aggressive metastases evade immune surveillance through lactate secretion, while latent lesions maintain redox homeostasis by relying on oxidative glutamine metabolism and the anion amino acid transporter (133). Notably, the brain’s distinctive low-lipid environment compels metastatic cells to enhance fatty acid synthesis, creating a synthetic lethal dependence on FASN (fatty acid synthase)—the key enzyme catalyzing *de novo* lipogenesis (134). This metabolic reprogramming may be regulated by brain-derived small extracellular vesicles (135). Brain and lung-derived sEVPs from tumor-bearing individuals show unique protein compositions that induce tumor cell DHFR expression and enhance metastatic potential (135).

Regarding microenvironmental adaptation, DTCs establish site-specific interactions with the neurovascular unit (136), and astrocytes maintain vascular-perivascular anchored DTCs in dormancy by secreting laminin-211 to block YAP nuclear translocation (136). This equilibrium can be disrupted by tumor-associated macrophage and microglia activation (137, 138). While CSF1R inhibition temporarily controls metastatic growth, established brain metastases trigger compensatory pro-inflammatory TAM activation through CSF2Rb-STAT5 signaling (137). Circulating tumor cell (CTC) studies have elucidated brain metastasis initiation mechanisms (139). Semaphorin 4D promotes blood-brain barrier penetration, while MYC expression proves critical for brain microenvironment adaptation. These discoveries provide new directions for developing therapeutic strategies targeting the specialized brain metastatic niche.Beyond the brain, bone and liver represent two additional organ sites with highly specialized metastatic niches. Despite their distinct microenvironments, both sites share common themes of immune suppression and metabolic adaptation, as discussed in the following section.

### 6.3 Bone and liver metastasis

Bone and liver represent two major distant metastatic sites for breast cancer, each with distinct microenvironmental features that impose unique selective pressures on disseminated tumor cells. While bone metastases are characterized by osteoclast-osteoblast remodeling and immunosuppressive myeloid infiltration, liver metastases involve interactions with hepatic stellate cells and unique metabolic adaptations. In this section, we first examine the molecular mechanisms driving bone metastasis, including the SCUBE2-Hedgehog axis and its impact on NK cell function. We then discuss liver metastasis, focusing on the role of activated hepatic stellate cells and neutrophil extracellular traps (NETs) in regulating tumor cell dormancy and migration. Understanding these organ-specific mechanisms is essential for developing targeted anti-metastatic therapies. In bone metastasis, ER-positive breast cancer drives osteoblast differentiation through the SCUBE2-Hedgehog axis, which subsequently suppresses NK cell function via collagen-LAIR1 signaling (140). Concurrently, the immunosuppressive microenvironment formed by granulocyte-T cell interactions is characterized by elevated IL1β expression and TIGIT/PD-1 signaling activation. *In vivo* targeting of these pathways can reactivate antitumor immunity, thereby reducing bone metastasis burden and improving survival rates (141).

Liver metastasis studies reveal that the interaction between NK cells and activated hepatic stellate cells (aHSCs) regulates DTC dormancy (142), while NETs mediate cancer cell migration through the CCDC25 receptor (143). These findings demonstrate the dual regulatory mechanisms of organ-specific metastasis, on one hand, through specific signaling pathways such as SCUBE2-Hedgehog (140) and CCDC25-ILK-β-parvin (143) that remodel the target organ microenvironment, on the other hand, by establishing immunosuppressive niches such as the IL1β/TIGIT network (141) and aHSCs-CXCL12 axis (142). Combined intervention strategies targeting these organ-specific mechanisms, such as simultaneously inhibiting SCUBE2 and CCDC25, or combining IL1β immunotherapy with NET inhibitors, may provide novel therapeutic approaches for advanced breast cancer.

**Figure 2:**
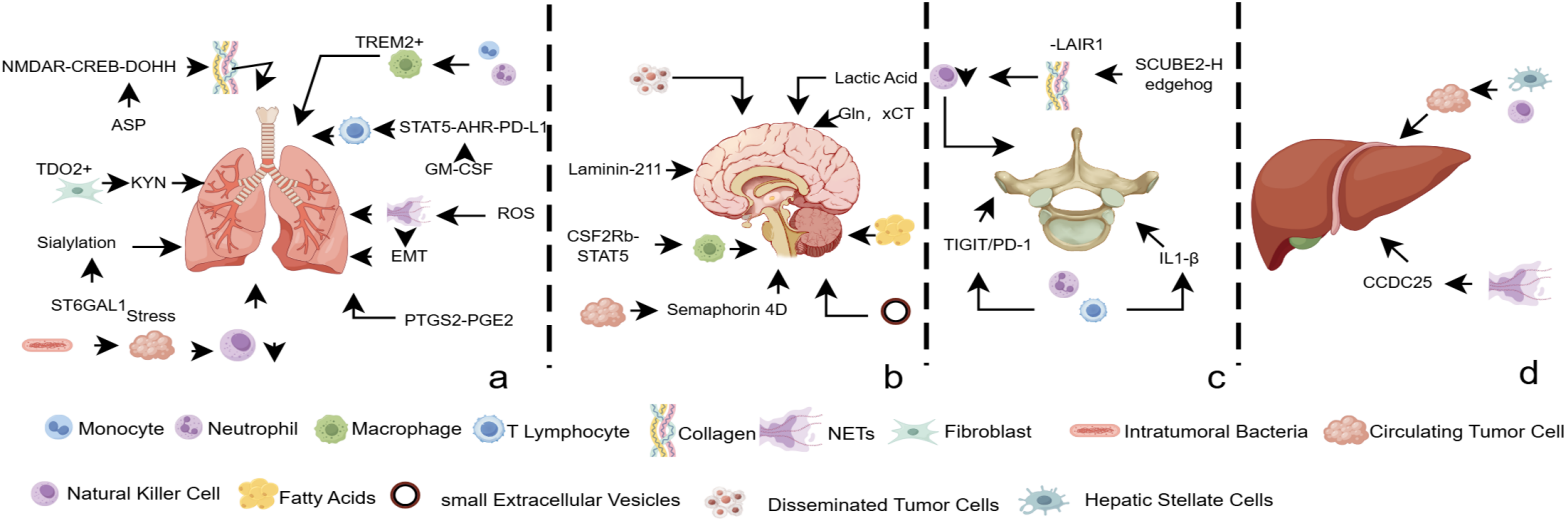
Distinct landscape of immune–metabolic–spatial tri-dimensional interactions in metastatic lesions. Systematic comparison of the unique adaptive alterations in immune suppression, metabolic reprogramming, and spatial architecture among breast cancer lung, brain, bone, and liver metastases highlights the site-specific microenvironmental signatures. **Figure 2a**. Lung metastasis. Three-dimensional synergy among immune suppression (TREM2^+^ macrophages, GM-CSF, NETs, PTGS2–PGE_2_ axis), metabolic adaptation (aspartate–NMDAR signaling, TDO2–KYN pathway, sialylation), and spatial remodeling (stress-induced CTC clusters). **Figure 2b**. Brain metastasis. Tri-dimensional coordination of immune regulation (TAMs), metabolic rewiring (lactate, glutamine, fatty-acid metabolism, sEVPs), and spatial reorganization (DTCs, laminin-211, CTC-derived semaphorin 4D). **Figure 2c**. Bone metastasis. Coordinated interplay between immune modulation (granulocytes, T cells, NK cells) and spatial restructuring (NETs). **Figure 2d**. Liver metastasis. Site-specific regulation through spatial restructuring (NETs-CCDC25 mediated migration), immune modulation (NK cell–hepatic stellate cell interaction regulating dormancy). Detailed molecular mechanisms are provided in Section 6. ROS, Reactive Oxygen Species; EMT, Epithelial-Mesenchymal Transition; KYN, Kynurenine; ASP, Aspartic Acid; Gln, Glutamine; xCT, cystine/glutamate antiporter.

Breast cancer metastasis is an organ-selective process shaped by the interplay of immunity, metabolism, and microenvironmental interactions. Research demonstrates that metastatic niches exhibit high specificity: lung metastasis depends on TREM2+ macrophages (121) and aspartate metabolic reprogramming (78), brain metastasis displays unique FASN dependency (134), and astrocyte-mediated dormancy regulation (136). Bone and liver metastases involve the SCUBE2-Hedgehog pathway (140) and NETs-CCDC25 migration axis (143). These discoveries reveal the three-dimensional regulatory network of metastatic lesions and highlight the therapeutic potential of targeting organ-specific microenvironments, including immunosuppressive niches, metabolic adaptation mechanisms, and spatial heterogeneity, providing new directions for developing precise anti-metastatic strategies (**Figure 2**).

## 7 Clinical translation

The translation of the immune-metabolic-spatial triad from mechanistic insights to clinical applications represents a critical frontier in overcoming breast cancer therapy resistance. While preclinical studies have established the rationale for multi-axis targeting, the clinical implementation of these strategies faces distinct challenges, including patient stratification, biomarker development, and trial design optimization. In this section, we first review ongoing and completed clinical trials that target one or multiple axes of the tripartite network, highlighting key findings and limitations. We then examine emerging drug development efforts that operationalize the three-dimensional framework through novel therapeutic agents and delivery systems. Finally, we discuss the future directions for intelligent delivery systems that may enable spatiotemporally coordinated multi-axis intervention.

### 7.1 Progress in current clinical trials

Clinical evaluation of therapies targeting the immune-metabolic-spatial triad has accelerated in recent years, with trials spanning multiple breast cancer subtypes and combinatorial approaches (55, 144–148). Here, we systematically summarize representative clinical trials, categorized by their targeted dimensions (**Table 1**), and analyze the emerging patterns of response across different therapeutic strategies and patient populations. This review systematically summarizes representative clinical trials from recent studies, categorized by their targeted dimensions (**Table 1**). These studies reveal that single-dimension immunotherapies, such as PD-1/PD-L1 inhibitors, show limited efficacy in certain subgroups (144, 149), likely due to the complexity of breast cancer-resistant microenvironments where isolated immune intervention fails to comprehensively disrupt immunosuppressive networks or effectively activate endogenous anti-tumor immune responses. In contrast, combination strategies incorporating metabolic modulation,e.g., apatinib (146), cromolyn sodium (38), or spatial targeting, e.g., CAR-M (16), anti-angiogenesis (150) demonstrate significantly improved responses. Notably, the cromolyn sodium plus PD-1 inhibitor trial (NCT05076682) (38) and the combination of sintilimab with anti-angiogenic agents (146) have clinically validated the feasibility of three-dimensional synergistic strategies, establishing new paradigms for overcoming therapeutic resistance.

**Table 1:** Summary of clinical trials classified by three-dimensional targeting of the breast-cancer drug-resistance microenvironment.

Targeting dimension	Trial name/design	Core intervention strategy	Key findings	Contribution to 3D theory	Breast cancer type	Reference
Immune	IMpassion031 (Phase III follow-up)	Atezolizumab neoadjuvant + chemotherapy	Atezolizumab group showed superior efficacy (HR=0.76)	Validation of long-term benefits of immunotherapy	TNBC	(149)
Immune	TREND (Phase II single-arm)	Tislelizumab + chemotherapy	pCR 68.18%, with ORR=93.18%	Demonstrates potential of immunotherapy combined with chemotherapy in TNBC	TNBC	(144)
Immune	IMpassion132 (Phase III RCT)	Atezolizumab + chemotherapy	PD-L1+ patients showed 40%↑ORR but no OS difference	Reveals limitations of single immune targeting	aTNBC	(143)
Immune	ER+ Immunotherapy (Phase III RCT)	Nivolumab + chemotherapy	PD-L1+ subgroup achieved pCR=44.3%	Shows positive significance of immunotherapy in ER+ BC	ER+/HER2-	(151)
Immune	GELATO (Phase II)	Atezolizumab + chemotherapy	TN-ILC clinical benefit rate 26%	Breakthrough in immunotherapy for triple-negative invasive lobular carcinoma	Invasive lobular carcinoma	(147)
Spatial+Immune	ATRACTIB (Phase II)	Atezolizumab + chemotherapy + bevacizumab	PD-L1- patients achieved mPFS 11.0 months	Vascular normalization enhances immune infiltration	aTNBC	(150)
Spatial+Immune	CT-0508 (CAR-M Phase I)	HER2-CAR-M therapy	HER2 3+ disease control rate 44%	Macrophage spatial reprogramming	HER2+	(28)
Metabolic+Immune	WSG-ADAPT-TN (Phase II Clinical Trial)	Anthracycline-free chemotherapy	Immune-related genes predicted pCR (AUC=83%)	Metabolic pathway genes correlate with survival; immune cell recruitment is significantly associated with pCR	eTNBC	(148)
Spatial+Immune+Metabolic	NCT05076682 (Phase II)	Cromolyn sodium + PD-1	ORR 50%; apMCs are mainly located in tertiary lymphoid structures	Highlights the spatial dimension importance in immunotherapy	TNBC	(49)
Metabolic+Immune+Spatial	NeoSAC (Phase II)	Sintilimab + apatinib + chemotherapy	pCR 70.6%; immune and oxidative stress scores predicted efficacy	Innovative breakthrough in understanding immune-metabolic-spatial interactions in TNBC resistance	TNBC	(145)

Importantly, distinct breast cancer subtypes, including TNBC (38, 144–146, 148–150), HER2+ breast cancer (16, 55), ER+/HER2- breast cancer (151), and invasive lobular carcinoma (147), exhibit markedly different responses to three-dimensional targeting strategies. This observation underscores the necessity to develop subtype-specific therapeutic frameworks for more precise and effective treatments. Current priorities include large-scale phase III validation of three-dimensional combination strategies, particularly for stage-specific precision matching (35, 50). Looking forward, developing predictive models integrating transcriptomic, metabolomic, and spatial multi-omics data (38, 145, 148) promises to identify key regulatory nodes within this complex three-dimensional interaction network. This innovative approach will provide a scientific foundation for developing more personalized and precise breast cancer treatment strategies. The combination strategies summarized above are not arbitrary pairings but are grounded in distinct mechanistic logics that emerge from the tripartite framework. Three principal rationales underlie the selections discussed in this review:

1. Removing parallel resistance barriers. Some combinations pair agents that target non-overlapping resistance mechanisms operating simultaneously within the same tumor. For example, PD-1 inhibitors combined with cromolyn sodium (targeting antigen-presenting mast cells) (37) address both T cell exhaustion and inadequate T cell priming—two immunologically distinct barriers that coexist in anti-PD-1 refractory TNBC.

2. Disrupting cross-axis dependencies. Other combinations target nodes where one axis directly sustains another. The addition of anti-angiogenic agents to immunotherapy (142, 146) exemplifies this logic: vascular normalization (spatial) relieves hypoxia (metabolic), which in turn restores T cell function (immune) by reducing lactate-mediated suppression and improving immune cell infiltration.

3. Preempting compensatory adaptation. A third rationale involves anticipating and blocking the escape pathways activated by single-axis targeting. PARP inhibitor combinations with CSF-1R blockade (82) illustrate this approach: PARP inhibition drives metabolic reprogramming in macrophages (metabolic adaptation), which is then preemptively countered by CSF-1R inhibition (immune targeting) to prevent the emergence of a immunosuppressive niche.

These mechanistic categories—parallel barrier removal, cross-axis dependency disruption, and compensatory adaptation preemption—provide a framework for rational combination design. They also explain why certain pairings succeed while others fail: effective combinations must align with the specific resistance architecture of the tumor subtype and treatment context, a principle that guides the ongoing trials discussed in this section.

While clinical trials validate the feasibility of multi-axis targeting, the development of novel therapeutic agents that operationalize this framework represents the next critical step. The following section examines emerging drugs and delivery systems designed to concurrently modulate immune, metabolic, and spatial dimensions.

### 7.2 Advances in drug development

The conceptual framework of immune-metabolic-spatial crosstalk has inspired a new generation of therapeutic agents designed to simultaneously disrupt multiple resistance nodes. These include immune-activating compounds, metabolic modulators, and spatially targeted delivery systems, as well as combination agents that integrate multiple functionalities. In this section, we highlight representative examples of these innovative therapeutics and discuss their mechanisms of action within the tripartite framework. In immune activation, DOX@3D-MPs significantly enhance anti-PD-1 efficacy by inducing immunogenic cell death and HSP70-mediated antigen presentation (152). For metabolic modulation, LDH inhibitor nanocomposites developed by researchers remodel the immunosuppressive microenvironment through targeted lactate metabolism intervention (73). Simultaneously, the gramine-platinum (IV) prodrug enhances anti-tumor immune responses by regulating the TGF-β/cGAS-STING metabolic-immune interaction pathway (153). In spatial delivery technologies, NExT biomimetic particles utilize exhausted T cell membrane-derived natural PD-1 receptors to achieve dual functions of precise drug delivery and immune checkpoint blockade (154), while XCSgel hydrogels overcome physical barriers by improving cytokine spatial retention to activate systemic anti-tumor immunity (155).

These innovative therapies have established triple-action mechanisms. NExT nanoparticles enhance immune recognition through PD-1/PD-L1 interaction (154), galloflavin nanocomposites improve metabolic microenvironment via LDH inhibition (73), and xanthoceraside-platinum(IV) complexes (GP) achieve spatial targeting through tumor-specific distribution (153). Together, they constitute an integrated immune-metabolic-spatial three-dimensional synergistic therapeutic strategy.

Future development of intelligent delivery systems will focus on three key directions: multi-target synchronous regulation (e.g., trifunctional nanoparticles carrying PD-1 antibodies, LDH inhibitors, and collagenase), dynamic responsiveness (systems autonomously adjusting drug release timing based on microenvironmental pH, ROS or enzyme activity changes), spatiotemporal precision control (combining imaging guidance for localized delivery and systemic therapy integration). These breakthroughs will transform breast cancer treatment from “single-dimension intervention” to “three-dimensional coordinated regulation,” providing innovative solutions to overcome clinical drug resistance challenges.

### 7.3 Translational challenges and priorities

The translation of tripartite targeting from concept to clinic faces three interconnected challenges that require explicit consideration.

Distinguishing clinical evidence from preclinical promise. As emphasized throughout this review, multi-axis targeting strategies span a spectrum of translational readiness. While combinations such as PD-1 inhibitors with cromolyn sodium (37) or anti-angiogenic agents (142, 146) have advanced to Phase II/III trials with reported efficacy outcomes, others—including CS-GFLG-DAS polymers (97), neutrophil-based delivery systems (101), and DOX@3D-MPs (148)—remain at preclinical proof-of-concept stages. Clear differentiation between these categories is essential for accurate assessment of the field’s maturity and for guiding future investment.

Actionable biomarkers for patient stratification. Identifying patients likely to benefit from tripartite targeting remains a critical bottleneck. Emerging candidates span all three axes: immunologically, the presence of tertiary lymphoid structures (TLS) and MHC-II^+^ tumor cells predicts response to immunotherapy alone, whereas their absence necessitates combination with radiotherapy to induce spatial synergy (36); metabolically, ASS1 expression identifies tumors sensitive to purine synthesis inhibitors combined with immune checkpoint blockade (83); spatially, DDR1-mediated collagen alignment correlates with T cell exclusion and may serve as a predictive biomarker for matrix-disrupting agents (89). Beyond single biomarkers, spatial metrics—such as the ratio of proliferating CD8^+^TCF1^+^ T cells to MHCII^+^ tumor cells (99)—integrate multiple axes and may offer superior predictive power. Prospective validation of these candidates in ongoing trials will be critical.Beyond these axis-specific candidates, computational frameworks that integrate multi-omics data and network biology offer systematic approaches to biomarker discovery. For example, FUNMarker—a fusion network-based method—integrates physical protein interactions from seven distinct sources with gene expression, GO annotations, and known disease-associated genes to identify prognostic and heterogeneous breast cancer biomarkers (156). By first clustering patients to account for sample heterogeneity and then applying label propagation on a weighted fusion network, this approach identifies biomarkers with both high discriminative power for patient survival and strong biological interpretability, as demonstrated by enrichment of TP53, AKT1, BRCA1, and ESR1 across multiple datasets. Such network-based strategies complement axis-specific candidate biomarkers and provide a systems-level framework for patient stratification in tripartite-targeted therapies.

Innovative trial design and its real-world challenges. Adaptive trial designs, umbrella/basket protocols, and platform trials offer flexibility for evaluating multi-agent combinations. However, their implementation faces practical hurdles: patient stratification requires real-time multi-omics profiling that is not universally available; toxicity management becomes increasingly complex with concurrent targeting of immune, metabolic, and stromal pathways; and endpoint selection must balance traditional measures (e.g., PFS, OS) with mechanism-based biomarkers that may not directly translate to survival benefit. The GELATO trial (143) exemplifies these challenges, demonstrating that even biomarker-selected populations may show limited responses when spatial and metabolic barriers are not concurrently addressed.

Addressing these challenges requires a shift from breadth to depth: prioritizing the most mature, mechanistically validated targets—such as ENPP1 (108), DDR1 (89), and TAM-directed therapies (38)—for accelerated clinical translation, while systematically building evidence for emerging candidates through well-designed proof-of-concept studies. This focused approach will maximize the likelihood of translating the tripartite framework into meaningful clinical advances.

## 8 Conclusions and perspectives

**Figure 3:**
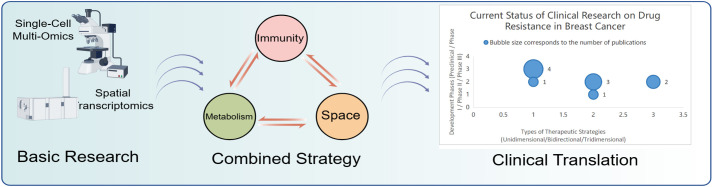
From basic research to combinatorial strategies and clinical translation: overcoming breast-cancer drug resistance. By integrating single-cell and spatial multi-omics to identify critical targets, and deploying immune–metabolic–spatial tri-dimensional combination strategies, we drive innovation in combination regimens during clinical translation to break the bottleneck of breast-cancer drug resistance. At present, no Phase III clinical trials simultaneously target the immune, metabolic, and spatial dimensions of breast-cancer drug resistance; closing this gap may become a pivotal focus for future research and a key breakthrough in conquering therapeutic resistance.

Breast-cancer drug resistance emerges from the dynamic interplay within a three-dimensional network, which includes immune suppression, metabolic reprogramming, and spatial heterogeneity. This intricate multidimensional cross-talk assembles a self-sustaining resistance niche that renders single-agent targeted therapy insufficient to overcome current therapeutic impasses. Recent work that systematically dissected these triaxial regulatory mechanisms has revealed the synergistic roles of immune-cell functional impairment, metabolite-mediated micro-environmental remodeling, and physical barriers that restrict drug penetration, thereby providing a robust theoretical foundation for the development of integrated clinical strategies (**Figure 3**).

At the immunological level, myeloid-derived suppressor cells—including M2-polarized TAMs and Tregs establish an immunosuppressive milieu through immune-checkpoint activation,e.g., PD-L1 and the secretion of inhibitory cytokines such as IL-10 and TGF-β (82, 88, 122). Metabolic reprogramming, achieved via hypoxia induction, glycolytic enhancement, and aberrant lipid metabolism, not only satisfies the energetic demands of tumors but also further cripples immune-cell function through lactate, reactive oxygen species, and other metabolites (56, 59). Spatial heterogeneity contributes by activating CAFs and remodeling the ECM, thereby erecting physical barriers and constructing specialized cellular niches that shield resistant subpopulations (66, 97). Importantly, these three dimensions do not operate independently; instead, they form a dynamic interactive network. Metabolic reprogramming modulates immune responses by altering immune-cell metabolic states, glycolytic flux, for example, inhibits T-cell infiltration, while spatial constraints modify the activity of metabolic enzymes by limiting drug distribution. Conversely, the immune micro-environment feeds back on metabolic pathways via cytokine secretion (73, 76, 108–110).

Targeting this complex network, three-dimensional combination regimens exhibit breakthrough potential. Immune-checkpoint blockade coupled with metabolic interventions,e.g., LDH or IDO inhibitors or stroma-directed agents, including anti-angiogenic drugs and CAF inhibitors, can disrupt the resistance circuit through multi-site synergism (71, 73, 100). Clinical validation is exemplified by the cromolyn sodium plus PD-1 inhibitor trial (NCT05076682) and the combination of sintilimab with anti-angiogenic agents (38). Furthermore, spatial-omics-guided precision strategies—such as DDR1-targeted disruption of collagen alignment or neutrophil-based drug-delivery platforms that traverse physical barriers—offer innovative solutions to overcome therapeutic resistance (92, 104).

Breast-cancer metastasis is not merely a passive dissemination of malignant cells but a systemic pathological process orchestrated by the immune–metabolic–spatial triad, endowed with pronounced selectivity and organ specificity. In pulmonary metastasis, remodeling of the immune micro-environment,e.g., TREM2^+^ macrophage enrichment, metabolic adaptation, such as aspartate-driven collagen synthesis, and spatial reconfiguration, immune evasion of polyclonal CTC clusters, etc., collectively foster metastatic outgrowth (121, 127, 130). Brain metastases display unique metabolic plasticity, lactate secretion to evade immune surveillance, and FASN-dependent fatty-acid synthesis, together with micro-environmental interactions in which astrocytes sustain disseminated tumor cell dormancy (133, 134, 136). Bone and hepatic metastases achieve niche adaptation through organ-specific pathways such as the SCUBE2–Hedgehog axis and the NETs–CCDC25 migratory program, respectively (140, 143). These findings illuminate the three-dimensional regulatory networks underlying metastatic lesions and underscore the therapeutic potential of targeting organ-specific micro-environments—including immunosuppressive niches, metabolic adaptation mechanisms, and spatial heterogeneity—thereby providing new directions for precision anti-metastatic strategies. Clinical translation still confronts substantial challenges. Distinct breast-cancer subtypes—triple-negative, HER2-, ER+/HER2-, and invasive lobular carcinoma—exhibit markedly heterogeneous responses to triaxial targeting regimens, suggesting that the establishment of a specific treatment system for subtypes may be a research hotspot in the future (55, 149, 151).

Future investigations should prioritize large-scale phase III validation of three-dimensional combination strategies and develop predictive models that integrate transcriptomic, metabolomic, and spatial multi-omic data to pinpoint critical regulatory nodes within the immune–metabolic–spatial network. This triaxial perspective not only deepens our understanding of breast cancer resistance mechanisms but also charts a course toward individualized, precision-based therapies, offering the prospect of improving patient outcomes by systematically dismantling the resistance niche.

## Glossary

TME: tumor microenvironmen
Tregs: regulatory T cells
IL-10: Interleukin-10
TGF-β: Transforming Growth Factor-beta
PD-L1: programmed death-ligand 1
NK cell: natural killer cell
ROS: reactive oxygen species
ECM: extracellular matrix
CAF: cancer-associated fibroblast
TNBC: triple-negative breast cancer
CAR-M: chimeric antigen receptor macrophage
PICs: physical interactions between neutrophils and cancer cells
DC: dendritic cells
PD-1: programmed cell death protein 1
TCR-T: T cell receptor-engineered T cell
TH2: helper type 2 cell
VEGE: vascular endothelial growth factor
IFN-γ: interferon-gamma
HER2: human epidermal growth factor receptor 2
HLA-G: human leukocyte antigen-G
ER: estrogen receptor
apMCs: antigen-presenting mast cells
TAM: tumor-associated macrophage
BTIC: breast tumor-initiating cell
IDC: invasive ductal carcinoma
ILC: invasive lobular carcinoma
PDX: patient-derived xenograft
MDSC: myeloid-derived suppressor cell
HR: hormone receptor
EMT: epithelial mesenchymal transition
ADCs: antibody-drug conjugates
mPFS: median progression-free survival
OXPHOS: oxidative phosphorylation
PPP: pentose phosphate pathway
USP10: ubiquitin specific peptidase 10
FMD: fasting mimicking diet
IMC: imaging mass cytometry
ADCC: antibody dependent cell mediated cytotoxicity
LDH: lactate dehydrogenase
FL: flaxseed lignans
ENL: enterolactone
NAA: N-acetylaspartate
IMC: imaging mass cytometry
TLS: tertiary lymphoid structure
ITH: intratumoral heterogeneity
QCCs: quiescent cancer cells
FLN: fibrotic microenvironments
NET: neutrophil extracellular trap
EVs: extracellular vesicles
DTCs: disseminated tumor cells
CTC: irculating tumor cell
aHSCs: activated hepatic stellate cells.
